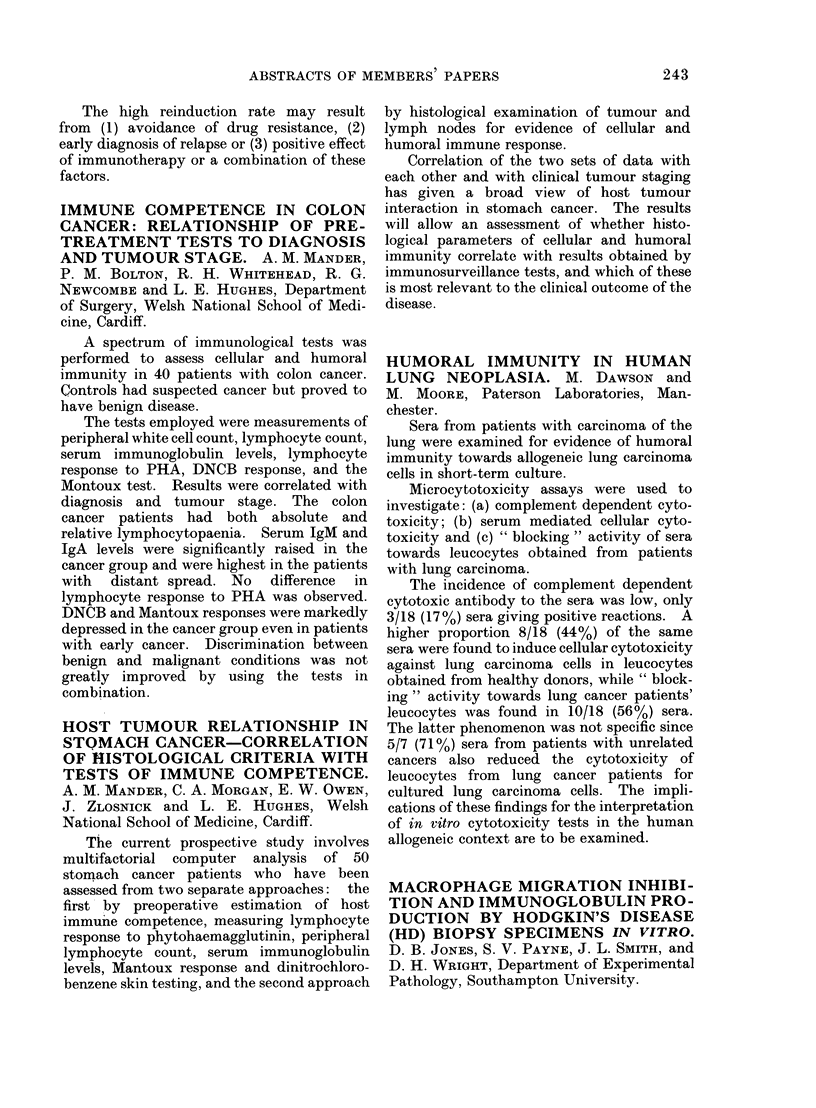# Proceedings: Immune competence in colon cancer: relationship of pretreatment tests to diagnosis and tumour stage.

**Published:** 1975-08

**Authors:** A. M. Mander, P. M. Bolton, R. H. Whitehead, R. G. Newcombe, L. E. Hughes


**IMMUNE COMPETENCE IN COLON CANCER: RELATIONSHIP OF PRE-TREATMENT TESTS TO DIAGNOSIS AND TUMOUR STAGE.** A. M. MANDER, P. M. BOLTON, R. H. WHITEHEAD, R. G. NEWCOMBE and L. E. HUGHES, Department of Surgery, Welsh National School of Medicine, Cardiff.

A spectrum of immunological tests was performed to assess cellular and humoral immunity in 40 patients with colon cancer. Controls had suspected cancer but proved to have benign disease.

The tests employed were measurements of peripheral white cell count, lymphocyte count, serum immunoglobulin levels, lymphocyte response to PHA, DNCB response, and the Montoux test. Results were correlated with diagnosis and tumour stage. The colon cancer patients had both absolute and relative lymphocytopaenia. Serum IgM and IgA levels were significantly raised in the cancer group and were highest in the patients with distant spread. No difference in lymphocyte response to PHA was observed. DNCB and Mantoux responses were markedly depressed in the cancer group even in patients with early cancer. Discrimination between benign and malignant conditions was not greatly improved by using the tests in combination.